# British Society of Echocardiography guideline for the transthoracic echocardiographic assessment of cardiac amyloidosis

**DOI:** 10.1186/s44156-023-00028-7

**Published:** 2023-08-31

**Authors:** William E. Moody, Lauren Turvey-Haigh, Daniel Knight, Caroline J. Coats, Robert M. Cooper, Rebecca Schofield, Shaun Robinson, Allan Harkness, David L. Oxborough, Julian D. Gillmore, Carol Whelan, Daniel X. Augustine, Marianna Fontana, Richard P. Steeds

**Affiliations:** 1 Queen Elizabeth Hospital Birmingham, University Hospitals Birmingham NHS Foundation Trust, Mindelsohn Way, Edgbaston, Birmingham, B15 2TH UK; 2 Institute of Cardiovascular Science, College of Medical and Dental Science, University of Birmingham, Birmingham, UK; 3 Division of Medicine, National Amyloidosis Centre, University College London, London, UK; 4 Queen Elizabeth University Hospital, Glasgow, UK; 5 Liverpool Heart and Chest Hospital, Liverpool, UK; 6 Liverpool John Moores University, Liverpool, UK; 7 North West Anglia Foundation Trust, Peterborough, UK; 8 Imperial College Healthcare NHS Trust, London, UK; 9 East Suffolk and North Essex NHS Foundation Trust, Essex, UK; 10 Sports and Exercise Sciences, Liverpool John Moores University, Liverpool, UK; 11 Royal United Hospitals Bath NHS Foundation Trust, Bath, UK; 12 Department For Health, University of Bath, Bath, UK

**Keywords:** Cardiac amyloidosis, Echocardiography, Transthyretin amyloid cardiomyopathy, Light chain amyloid cardiomyopathy

## Abstract

These guidelines form an update of the BSE guideline protocol for the assessment of restrictive cardiomyopathy (Knight et al. in Echo Res Prac, 2013). Since the original recommendations were conceived in 2013, there has been an exponential rise in the diagnosis of cardiac amyloidosis fuelled by increased clinician awareness, improvements in cardiovascular imaging as well as the availability of new and effective disease modifying therapies. The initial diagnosis of cardiac amyloidosis can be challenging and is often not clear-cut on the basis of echocardiography, which for most patients presenting with heart failure symptoms remains the first-line imaging test. The role of a specialist echocardiographer will be to raise the suspicion of cardiac amyloidosis when appropriate, but the formal diagnosis of amyloid sub-type invariably requires further downstream testing. This document seeks to provide a focused review of the literature on echocardiography in cardiac amyloidosis highlighting its important role in the diagnosis, prognosis and screening of at risk individuals, before concluding with a suggested minimum data set, for use as an aide memoire when reporting.

## Key points

- Echocardiography has an important role in raising the suspicion of cardiac amyloidosis although a diagnosis cannot be made without confirmatory tests.
- Cardiac amyloidosis is relatively common and should enter the differential diagnosis for any patient presenting with increased left ventricular wall thickness.
- Performing global longitudinal strain (GLS) is important for the assessment of *any* patient presenting with increased left ventricular wall thickness.
- Amyloid fibrils infiltrate the valves and the atria, as well as the ventricular myocardium.
- GLS and E/e’ have a high probability of being abnormal in the early stages of cardiac amyloidosis
- Asymmetric wall thickening does *not* exclude a diagnosis of cardiac amyloidosis.
- GLS, stroke volume index and worsening degrees of mitral and tricuspid regurgitation are important predictors of prognosis in cardiac amyloidosis.

## Overview of cardiac amyloidosis

Amyloidosis is not a single disease entity but a collection of disorders characterized by protein misfolding. When these insoluble proteins accumulate as fibrils in the myocardial interstitium (the extracellular space), this results in increased myocardial wall thickness (pseudo-hypertrophy) and produces a restrictive cardiomyopathy. Almost all forms of cardiac amyloidosis (circa. 95%) result from two subtypes: (1) AL, where the precursor protein is an abnormal immunoglobulin light chain and, (2) ATTR, where the precursor protein is transthyretin (TTR) .

### Light chain amyloidosis

In AL amyloidosis, the light chains are usually produced by abnormal plasma cells in the bone marrow. These aggregate to form insoluble fibrils, which deposit in tissues and cause organ dysfunction. The heart is the most commonly involved organ in AL amyloidosis with > 75% of patients at diagnosis exhibiting symptoms from light chain amyloid cardiomyopathy (AL-CM) [2, 3]. Median age at diagnosis is 65 years [4]. The initial symptoms are often non-specific (e.g. weight loss and fatigue) which can result in delays of > 6 months before the diagnosis is made, by which time patients have commonly seen several specialists [5]. An estimated 10–15% of AL amyloidosis cases occur in association with multiple myeloma. The circulating free light chains are toxic to myocytes. The median survival, from diagnosis, of patients with advanced AL-CM is only 4 months and approximately 2 years in patients with less severe cardiac involvement [4]. Although the introduction of new therapeutic regimens has improved the overall survival of patients, treatment is still poorly tolerated among patients with cardiac and renal involvement.

### Transthyretin amyloidosis

Transthyretin is primarily synthesized in the liver and acts as a serum transport protein for thyroxine and retinol. ATTR amyloidosis is subdivided by the sequence of the *TTR* gene into wild-type (wtATTR) or hereditary (hATTR). The former is primarily seen in older patients (median age at diagnosis 79 years) with a strong male predominance [6], while hATTR tends to present earlier as an autosomal dominant disorder resulting from pathogenic heterozygous variants in the *TTR* gene. Although more than 130 amyloidogenic *TTR* variants have now been identified worldwide, the two which are responsible for the majority of disease-causing hATTR cardiac amyloidosis in the UK and Ireland are the V142I (formerly V122I) and T80A (formerly T60A) variants [7]. Patients with wild-type transthyretin amyloid cardiomyopathy (wtATTR-CM) typically present with symptoms of heart failure often associated with peripheral oedema, conduction disturbances and frequently, with a preceding history of atrial arrhythmia. As with AL amyloidosis, initial symptoms are non-specific. The diagnosis of ATTR-CM is often made several years after symptom onset and considered only after multiple hospital admissions [6]. Recent data suggest, however, that advances in cardiac imaging (echocardiography, bone scintigraphy and cardiac MRI) are enabling earlier diagnosis, which is associated with improved outcomes [7].

The two main subtypes of cardiac amyloidosis represent very different disease processes with significant heterogeneity in clinical course, prognosis, and management. AL amyloidosis is characterized by a rapidly progressive clinical course. In contrast, hATTR amyloidosis follows a more variable clinical course depending upon the specific variant inherited with either cardiomyopathy and/or sensory and autonomic neuropathy. Furthermore, hATTR amyloidosis is characterized by an age-dependent penetrance. The clinical phenotype in wtATTR-CM is of a much slower but nonetheless progressive and eventually fatal cardiomyopathy, with a median survival of 5 years without access to disease modifying therapy [6]. Irrespective of ATTR subtype, early suspicion and prompt diagnosis of cardiac amyloidosis is key to targeting treatments and thereby improving patient outcomes; there is now evidence from randomized controlled trials that TTR directed therapies can provide benefit to subjects when treated early in the disease process [8–11]

In summary, while there are current delays to diagnosis in both AL and ATTR-CM, a key point is that skilled assessment by echocardiographers alert to the possibility of amyloid will lead to early diagnosis and treatments with the potential to improve quality and length of life for the patient.

## The changing epidemiology of cardiac amyloidosis

A contemporary systematic review and meta-analysis of 31 screening studies, provides increasing support for the notion that cardiac amyloidosis, and in particular wtATTR-CM, should no longer be considered a rare condition [12]. As many as 1 in 8 patients with severe aortic stenosis (AS) referred for transcatheter aortic valve implantation (TAVI) had evidence of amyloid based on 99mTc-DPD scintigraphy [13], while combining registry data from heart failure with preserved ejection fraction (HFpEF) cohorts supported a similarly high pooled prevalence (12%) [14, 15]. It is also notable that cardiac amyloid can easily mimic hypertrophic cardiomyopathy (HCM): there was a pooled prevalence of 7% of cardiac amyloidosis among adult patients referred to tertiary centres with an original diagnosis of HCM [16, 17].

Registry data from 11,006 UK patients who received a diagnosis of amyloidosis in the period 1987–2019, suggests that AL amyloidosis remains the most common type, accounting for 55% of all cases. The diagnosis of wtATTR-CM has however, increased exponentially from a referral prevalence of < 3% in the period 1987–2009 to 25% between 2016 and 2020 [18]. The true prevalence of cardiac amyloidosis is still debated, and the published figures will vary depending on the cohort being actively screened. Irrespective, ATTR-CM is increasingly recognized as an emerging cause of heart failure morbidity and mortality worldwide. A comprehensive review which further examines the pathogenesis, epidemiology, diagnosis, and treatment of ATTR-CM related to its wild-type and hereditary forms, has recently been published [19].

## Characteristic echocardiographic features of cardiac amyloidosis

The original reports describing the hallmark “stiff heart” echocardiographic findings of cardiac amyloidosis were published in the mid 1970s [20, 21]. Since then, echocardiography has become a routine part of the diagnostic and prognostic assessment of patients with suspected or confirmed cardiac amyloidosis. It is worth remembering that amyloid fibrils deposit throughout the heart (valves, atrial walls, and ventricular myocardium) but the process is usually most evident in the ventricular walls.

### Increased wall thickness

The hallmark feature of cardiac amyloid deposition is increased left ventricular (≥ 12 mm) or biventricular wall thickness in the absence of aortic valve disease, significant systemic hypertension or another plausible cause. It is worth acknowledging, however, that cardiac amyloidosis and AL-CM in particular, can present with relatively minimal increase in LV wall thickness and a normal LV mass. The increase in wall thickness is a form of ‘pseudohypertrophy’ caused by progressive infiltration of amyloid in the extracellular space (i.e. the interstitium) rather than a reflection of cardiomyocyte hypertrophy [22]. This is the mechanism underlying the characteristic discrepancy between small, low voltage QRS complexes on ECG and increased wall thickness on echocardiography, a finding which can be very helpful in discriminating cardiac amyloidosis from HCM [23].

### Restrictive physiology

**Fig. 1:**
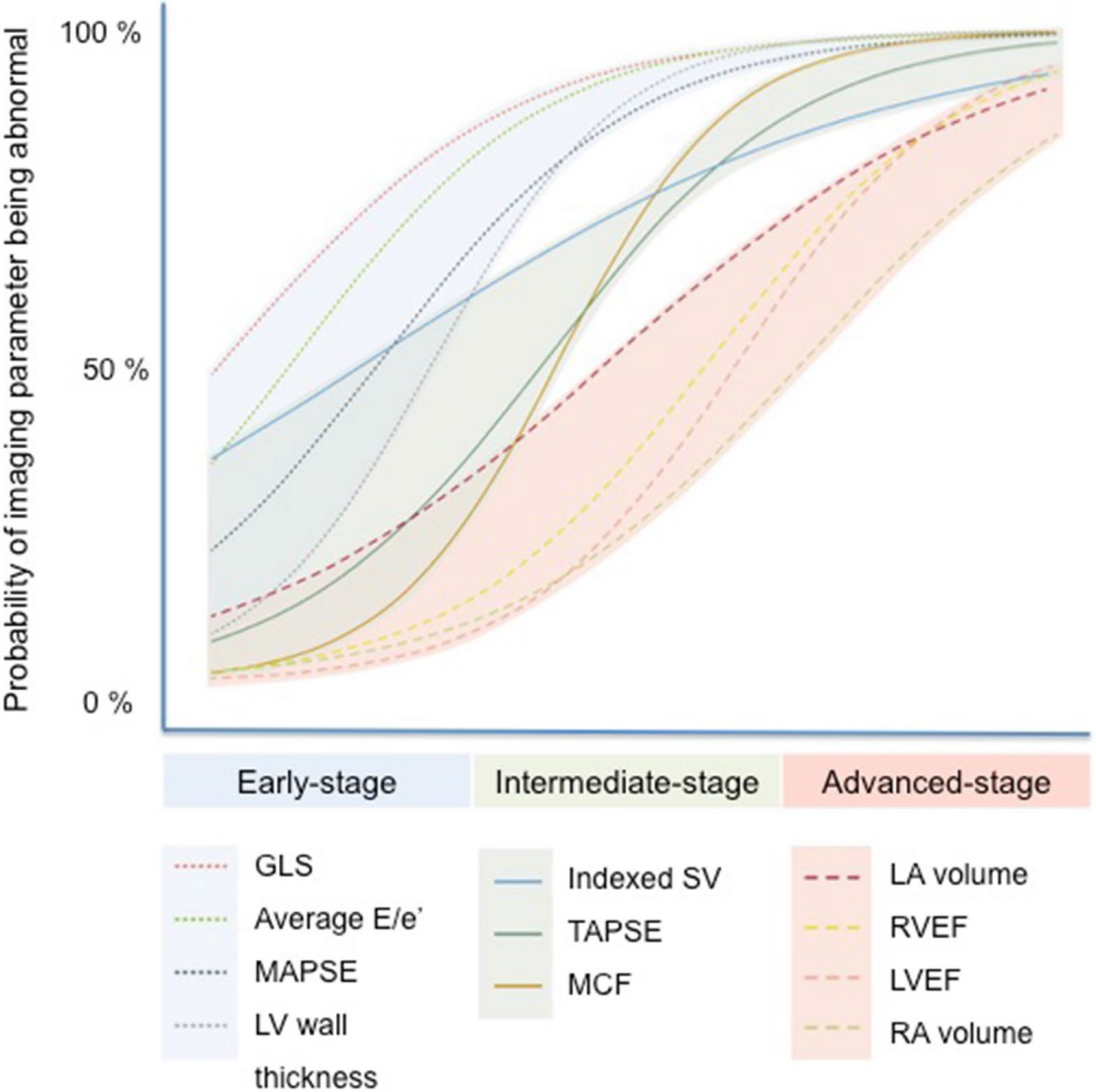
Probability of echocardiographic variable being abnormal according to the amyloid disease burden. Adapted from Knight et al. JACC 2019 [28]

The other classical echocardiographic features of cardiac amyloidosis include a normal or small LV cavity size, a restrictive transmitral Doppler filling pattern related to stiff, non-compliant ventricles with poor longitudinal systolic function. It is also often associated with bi-atrial dilatation, raised filling pressures, left atrial stasis with spontaneous echo contrast, interatrial septal thickening, valve thickening and the presence of pericardial and pleural effusions [24, 25]. The majority of these findings in isolation have a low accuracy for diagnosing cardiac amyloidosis, mostly due to low sensitivity. For example, a clear-cut restrictive mitral inflow pattern is rarely seen until the late stages of the disease, but early diastolic dysfunction [26] and signs of raised filling pressures may be among the first echocardiographic signs of early amyloid infiltration and provide greater sensitivity (Fig. 1) [27, 28].

### Tissue Doppler imaging and speckle-tracking echocardiography

Performing Tissue Doppler imaging (TDI) and speckle-tracking echocardiography (STE) to quantitate longitudinal systolic function in all patients with increased wall thickness or left ventricular hypertrophy is critical. Combining techniques can help discriminate between other potential causes of LV wall thickening and HFpEF [29–31]. For example, a pattern of normal LV ejection fraction (EF) despite diminished longitudinal systolic function, is typical of cardiac amyloidosis and can help differentiate it from early sarcomeric HCM in which LV EF is more often high or “supra-normal” rather than normal [32]. Longitudinal systolic function is impaired in cardiac amyloidosis even in the earliest phase of the disease [25]. More importantly, both AL and ATTR cardiac amyloidosis patients demonstrate a characteristic ‘bulls-eye’ pattern of apical sparing on longitudinal strain imaging, which is sensitive (93%) and specific (82%) permitting discrimination of amyloidosis from others causes of LV wall thickening [33] and also portends an adverse prognosis [28]. Conversely, patients with other causes of LV hypertrophy (e.g., aortic stenosis, HCM) more typically exhibit reduced LV longitudinal strain in the regions of maximal hypertrophy. The mechanism underlying the apical sparing pattern in amyloidosis is debated but less amyloid deposition at this level relative to the base is one plausible explanation [32]. Given the sensitivity and relatively high specificity of this imaging finding compared to other echocardiographic parameters, it is recommended that all patients with increased LV wall thickness should undergo strain imaging, not only those patients suspected of having cardiac amyloidosis. This is especially important because raising a suspicion of amyloidosis and facilitating an early diagnosis permits timely treatment of patients, thereby improving prognosis.

### Relative utility of echo parameters

A plethora of echocardiographic findings have been suggested for distinguishing cardiac amyloidosis from other causes of increased myocardial wall thickness (Table 1). In a small study (n = 100) including patients with cardiac amyloidosis, HCM and hypertensive heart disease, the EF to global longitudinal strain (GLS) ratio (EFSR) was reported as the single best discriminating deformation-based parameter [34]. Based on a much larger echocardiography study of patients with ATTR-CM (n = 1240), myocardial contraction fraction (MCF), the ratio of stroke volume to myocardial mass, is the parameter with the highest diagnostic accuracy (AUC 0.80) [32]. Over time, progressive amyloid deposition into the myocardium results in increasing myocardial mass and a progressively smaller ventricular cavity size with low stroke volume [32]. This mechanism results in a state of fixed end-diastolic volume where cardiac output then becomes critically dependent on heart rate. The situation is exacerbated further by amyloid infiltration into the valves which results in mitral and tricuspid regurgitation further limiting forward stroke volume [35].

**Table 1:** Relative utility of echo parameters for diagnosis and prognosis in cardiac amyloidosis

Echo parameter*	ATTR-CM		AL-CM	
	Diagnosis	Prognosis	Diagnosis	Prognosis
LV wall thickening	+ +	+	+	+
RV wall thickening	+ +	+	+ +	+
Pericardial effusion	+	+	+ +	+ +
Apical sparing GLS pattern	+ + +	NA	+ + +	NA
Reduced GLS	+	+ + +	+	+ + +
Elevated E/e′	+ +	+ +	+ +	+ +
Bi-atrial dilatation	+ +	+ +	+ +	+ +
Low-flow low-gradient severe AS	+ +	+	NA	NA
RV systolic dysfunction (FAC, RV s′)	NA	+ + +	NA	+ + +
Mitral regurgitation (Carpentier Type 1 / 3a)	+ +	+ + +	+	+ +
Tricuspid regurgitation (Carpentier Type 1 / 3a)	+ +	+ + +	+	+ +
EFSR**	+ + +	NA	+ + +	NA
MCF ratio**	+ + +	+	+ + +	+

*AL-CM* light chain amyloid cardiomyopathy, *ATTR-CM* transthyretin amyloid cardiomyopathy, *EFSR* left ventricular ejection fraction to global longitudinal strain ratio, *FAC* fractional area change, *GLS* global longitudinal strain, *LV* left ventricular, *MCF* myocardial contraction fraction ratio, *NA* not applicable, *RV* right ventricular

^*^The diagnostic specificity of all echo parameters is reduced in the context of uraemic or hypertensive cardiomyopathy

^**^These echo parameters provide diagnostic utility in cardiac amyloidosis but are not included in the BSE minimum dataset

Although it has not yet been externally validated, a proposed echocardiographic scoring system using specific echocardiographic parameters has been endorsed by the European Society of Cardiology to aid with the diagnosis of cardiac amyloidosis [36, 37]. In patients with LV wall thickening, an increased wall thickness (IWT) score calculated using parameters including relative wall thickness (RWT), E/e′, TAPSE, GLS, and septal longitudinal systolic apex-to-base strain ratio (SAB), provides a high diagnostic accuracy for ATTR-CM (≥ 8 points provided an AUC of 0.87) [36]. In patients with systemic AL amyloidosis, an AL score of ≥ 5 points based on parameters including RWT, E/e′, GLS, and TAPSE, also showed very good diagnostic accuracy in identifying patients with AL-CM with an AUC of 0.90. There is also growing interest in machine learning tools to help differentiate causes of increased LV wall thickness [38, 39]. This strategy may prove more successful than scoring systems based on regression analysis of data from subjects referred to a national centre with a suspicion of amyloidosis [37], when the aim is to identify patients with amyloidosis from a wider cardiology population.

## Potential pitfalls

Of note, the threshold maximal wall thickness of ≥ 12 mm is not sex-specific and, if used in isolation without taking into account a historical blood pressure profile, confers a high sensitivity, but a low specificity for the diagnosis of cardiac amyloidosis. For this reason, the assessment of patients with increased wall thickness should be performed in combination with the other echocardiographic findings highlighted above. Clinicians in particular, must interpret the findings of the echocardiogram in the context of other clinical red flags such as a history of bilateral carpal tunnel syndrome [40].

A granular speckling appearance of the myocardium has long been described in amyloidosis. This term should be avoided, however, due to its lack of specificity; as well as being seen in cardiac amyloidosis, “speckling” has been described in numerous other conditions, including hypertensive heart disease, chronic kidney disease, HCM and Pompe’s disease [41]. Moreover, the shift for routine echo acquisition from fundamental to harmonic imaging has further confounded this observation [40].

## Asymmetric versus concentric remodeling

Including measurement of relative wall thickness (RWT) is important and dichotomises left ventricular remodelling/hypertrophy as concentric (> 0.42) or eccentric (≤ 0.42) based on agreed cut-offs. This derived parameter performs part of the IWT and AL scores which have been endorsed by the European Society of Cardiology but should be viewed with caution [36, 37]. Patients with cardiac amyloidosis have historically been thought to develop concentric remodelling, which is usual in AL-CM but many patients with ATTR-CM develop increased wall thickness with asymmetric septal predominance [42, 43]. In a cardiac MRI study including 263 ATTR-CM patients, 79% of patients demonstrated asymmetrical hypertrophy while only 18% were classified as having concentric wall thickening defined as a septal:lateral wall thickness ratio < 1.5. While the majority of patients with a wall thickness ratio of > 1.5 had a sigmoidal septal pattern of remodelling (55%), nearly a quarter of patients with ATTR-CM (24%) had a reverse septal contour– a morphology which traditionally has been regarded as characteristic of HCM [23, 42]. Thus, a key point is that asymmetry does *not* exclude cardiac amyloidosis.

Relative wall thickness has been measured in clinical studies both as: RWT = 2 * PWd / LVIDd and RWT = IVSd + PWd / LVIDd. Given the propensity for cardiac amyloidosis to cause asymmetric increase in septal wall thickness, the calculation of RWT can be underestimated when only posterior wall thickness is used. Although these formulae have in the past been used interchangeably, for this reason, the BSE supports use of the formula that includes septal wall thickness:

RWT = IVSd + PWd / LVIDd.

## Phenocopies and limitations of echocardiography

**Fig. 2:**
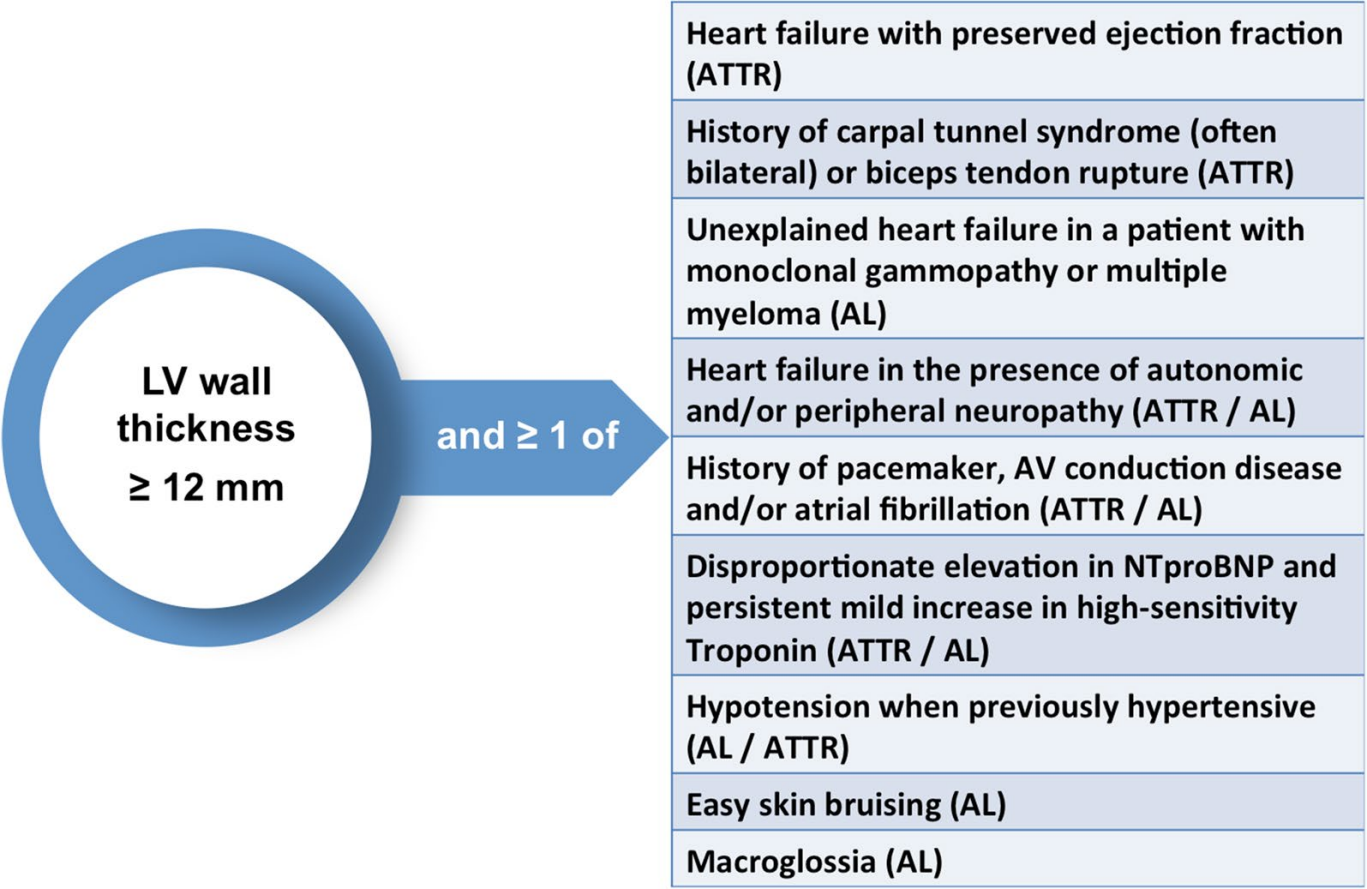
When to suspect a diagnosis of cardiac amyloidosis? AL, amyloid light chain, ATTR, transthyretin amyloid, AV, atrio-ventricular. Adapted from the ESC position statement [37]

**Fig. 3:**
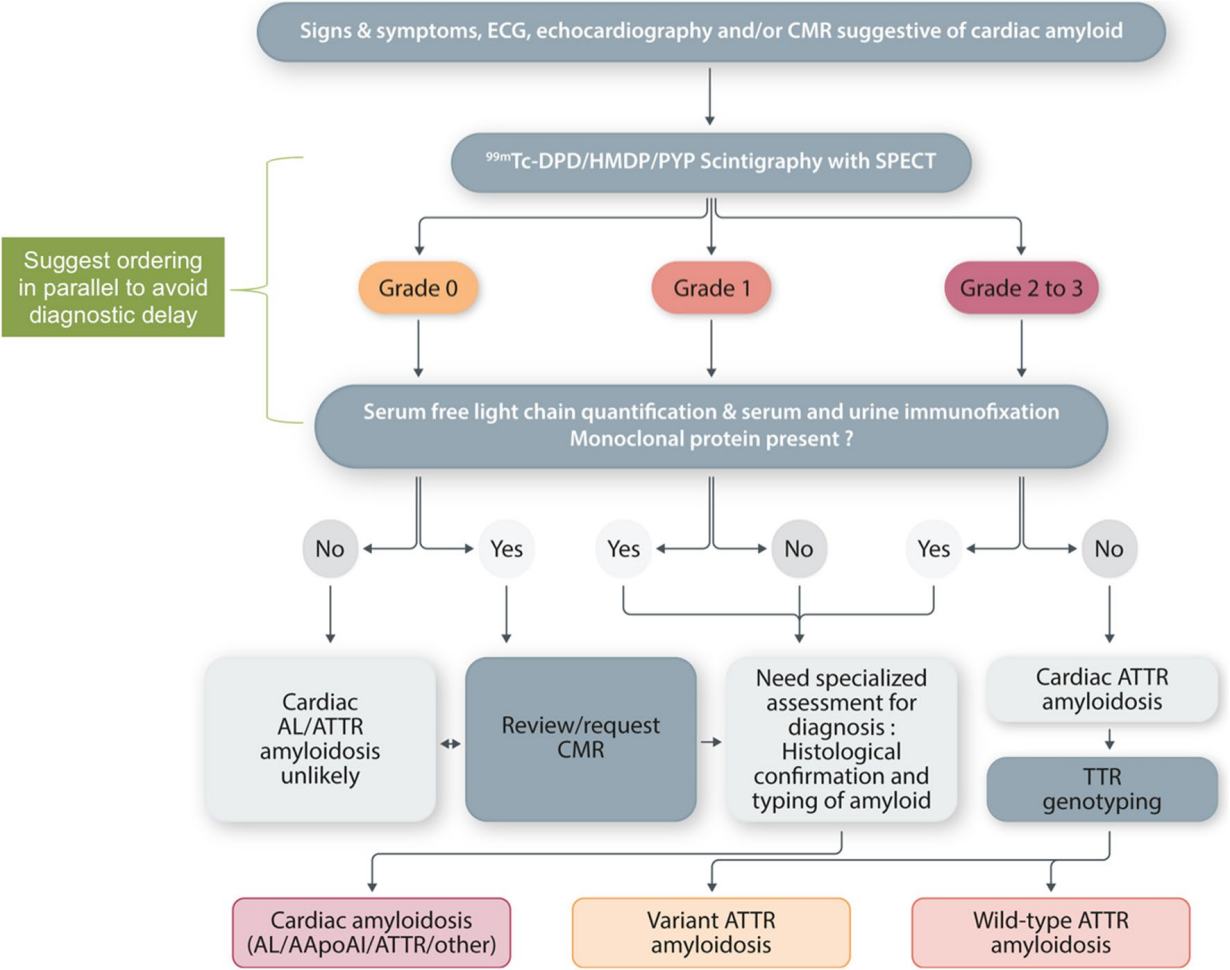
Diagnostic algorithm for patients with suspected cardiac amyloidosis. 99mTc-DPD, 99mTc-3,3-diphosphono-1,2-propanodicarboxylic acid; 99mTc- HMDP, 99mTc-hydroxymethylene diphosphonate; 99mTc-PYP, 99mTc-pyrophosphate; AApoA1, apolipoprotein A-I; AL, light chain; CMR, cardiac magnetic resonance; SPECT, single-photon emission tomography imaging; TTR, transthyretin. Re-adapted with permission from Gillmore et al. [44]

Cardiac amyloidosis should enter the imaging differential diagnosis for any patient that presents with left ventricular wall thickening (Fig. 2). Ethnicity, hypertension, chronic kidney disease, significant aortic valve disease, obesity, diabetes mellitus and athletic remodeling will all influence the degree of LV wall remodelling. In particular, there will be “grey cases” when echocardiography cannot discriminate between HCM and cardiac amyloid [23]. In such patients, clinicians will need to review the echocardiography findings in the context of the medical history, any relevant family history, the ECG, and often perform downstream complementary imaging (Figs. 2 and 3). Whilst echocardiography is frequently able to raise the suspicion of amyloidosis as a disease, it is not possible to make the diagnosis and differentiate AL-CM from ATTR-CM by this imaging technique alone. Although, it is widely acknowledged that patients with ATTR amyloidosis tend to demonstrate greater degrees of LV wall thickening compared to those with AL amyloidosis, additional tests must be performed before amyloid can be sub-typed and a final diagnosis reached (Fig. 3) [44].

## Recommendations for reporting

The British Society of Echocardiography is not endorsing a scoring model [37] and recommends a likelihood-based report based on the imaging findings [40]. An overall interpretation of the echo findings is recommended into categories of:

1. Not suggestive: Normal LV wall thickness, normal atrial size, septal or lateral tissue Doppler e′ velocity > 10 cm/s, normal GLS and absence of apical sparing longitudinal strain pattern.
2. Equivocal: Mixed parameters.
3. Suggestive: Increased LV wall thickness, reduced GLS with typical apical sparing strain longitudinal strain pattern, restrictive Doppler mitral inflow pattern, restrictive mitral annular TDI.

Suggestive echocardiographic findings cannot distinguish AL-CM from ATTR-CM and indeed, there is considerable overlap between findings. On that basis, it is suggested that the likelihood of cardiac amyloidosis should be reported but no comment should be made regarding amyloid sub-type within echocardiography reports.

## Amyloid and low-flow low-gradient aortic stenosis

There is an important association between ATTR-CM and AS [13, 45]; and a high proportion of patients with amyloid associated with severe AS will present with parameters consistent with a low-flow low-gradient AS with LVEF > 50%. Most patients with cardiac amyloidosis present in a low cardiac output state with limited ability to increase their stroke volume; thus AV Vmax and mean gradient will often be low, particularly in the presence of concomitant mitral regurgitation and atrial fibrillation. No echocardiographic features can reliably differentiate amyloid patients with concomitant severe AS from those with severe AS without amyloid. Although reduction in GLS is not specific to amyloidosis, patients with amyloidosis will often have LV wall thickening which is disproportionate to the severity of AS. A prospective multi-centre study proposed a clinical scoring system which offers good discrimination (AUC 0.85) between lone AS and dual pathology amyloid-severe AS; this score did incorporate echo parameters (IVSd > 18 mm and E/A > 1.4) but was more strongly weighted towards non-echo findings (prior carpal tunnel syndrome and RBBB on ECG). The same cohort study provides support for aortic valve intervention in those patients with symptomatic severe AS and concomitant ATTR-CM [46].

## Serial echocardiography and determining prognosis

Until recently, there were only relatively limited data from small-scale studies on the utility of echocardiography in determining prognosis specific to cardiac amyloidosis [47–49]. A number of echocardiographic variables correlate strongly with extracellular volume (ECV), a CMR derived marker of amyloid burden within the myocardial interstitium which does provide robust prognostic information [50]. Based on cross-sectional data, GLS and E/e′ by echocardiography have a high probability of being abnormal at low ECV. Conversely, at higher cardiac amyloid burden, atrial areas and EF become abnormal [28].

In patients with ATTR-CM, baseline echo derived stroke volume index, GLS, E/e′ and right atrial area index, have all been independently associated with mortality [32]. Similarly, in AL-CM, baseline GLS and stroke volume index predict survival [51, 52]. Echo indices appear to provide incremental prognostic utility to cardiac biomarkers. Following chemotherapy, patients with AL-CM achieving an improvement in GLS and a reduction in N-terminal pro-brain natriuretic peptide survive longer than those patients achieving a reduction in this cardiac biomarker in isolation [51]. A recent large prospective cohort study of 565 wild-type ATTR-CM and 312 hereditary ATTR-CM patients, has also highlighted the prognostic importance of amyloid infiltrating the atrioventricular valves. In this study, among a wide range of echo indices including strain-based parameters, only worsening in the degree of mitral and tricuspid regurgitation was independently associated with adverse prognosis [35].

The optimal frequency of repeat echocardiography is not currently known and will be influenced by factors including the amyloid sub-type and clinical response to treatments. It is suggested that the treating specialist should, therefore, determine the timing and appropriateness of repeat studies.

## The role of echocardiography in the screening of at risk individuals

The importance of echocardiography in people without symptoms who may have hATTR amyloidosis (i.e. relatives identified with *TTR* variants) has recently been highlighted in a UK consensus statement guideline [7]. Alongside CMR and 99 m-Tc DPD imaging, echocardiography forms an integral part of the clinical surveillance of at-risk individuals and permits a functional assessment of pre-symptomatic carriers of *TTR* variants. Cardiac assessments should be offered within 10 years of the predicted age of onset relating to the phenotype associated with a specific *TTR* variant (age of onset typically > 50 years for the two commonest variants in the UK and Ireland: V142I (formerly V122I) and T80A (formerly T60A)).

## Minimum dataset

### Transthoracic echocardiography cardiac amyloidosis protocol

**Table:** 

Measurement	View	Modality	Explanation	Image
*Left ventricle (LV)*				
LV dimensions	PLAX view	2D Units: LVIDd mm LVIDs mm	LV dimensions are obtained from the parasternal long axis (PLAX) window preferentially using 2D imaging [53]. Freeze at end-diastole and measure the diameter of the interventricular, LV internal diameter and posterior wall in line with the MV leaflet tips. Repeat the LV cavity diameter at end-systole LV cavity is usually normal or small in size in cardiac amyloidosis[54]	
LV wall thickness measurements	SAX view: MV level Mid-LV level Apical level	2D Units: mm	Freeze 2D image at end-diastole. Caliper the diameter of maximal wall thickness wherever it occurs, in the septum, anterior, lateral and inferior walls at all three levels Be careful not to include right ventricular (RV) wall, papillary muscles, trabeculations or moderator band	
LV mass and relative wall thickness	PLAX	2D	LV wall thickness by itself does not define an individual as having left ventricular hypertrophy (LVH). Rather, the presence or absence of LVH is determined from LV mass after indexing to BSA. Wall thickness measurements, combined with the LV internal diameter in diastole, can be used to determine the RWT In the context of an increased mass, an RWT > 0.42 defines the pattern of LVH as being ‘concentric’. If the RWT is ≤ 0.42, the pattern of LVH is ‘eccentric’ [53]	
LV	A4C, A2C, A3C	2D	It is vital to acquire good quality echo images of the left ventricle in the apical windows Optimise images to increase frame rate and maximise image quality, to optimise quantification of strain. Do not comment on a granular speckling appearance of the myocardium	
LV Simpson’s Biplane volumes	A4C, A2C	2D Units: EDV mL ESV mL	LV volumes should be obtained using 2D imaging from A4C and A2C, and wherever possible 3D imaging Trace the endocardial border. LV length is defined as the distance between the midpoint of the mitral valve level line and the most distal point of the LV apex Measure at end-diastole (onset of QRS complex) and end-systole (the frame before MV opens, where AV just closes) Volumes are indexed to body surface area Take care to ensure the LV is not foreshortened. Papillary muscles and trabeculations are excluded from the volumes and considered part of the chamber A reduction in LV end-diastolic volume can often lead to a reduced stroke volume despite normal LVEF in the early stage of the disease. Estimation of stroke volume should also be included based on measurement of LV outflow tract diameter and the velocity–time integral (on pulsed wave Doppler)	
LV Simpson’s Biplane ejection fraction (EF)	A4C, A2C	2D Units: EF %	LVEF should be derived using the biplane Simpson’s method from 2D volumes, obtained from the apical 4- and 2-chamber views as described above. It is essential that values for LVEF are not derived from foreshortened or poorly obtained volumetric data [53]. EF may be reduced in end stages, but is more often normal in early disease	
Global longitudinal strain	A4C, A2C, A3C	2D Units: -%	Average global longitudinal strain (GLS) is calculated using the apical long axis (A3C), four chamber (A4C) and two chamber (A2C) standard views. High quality image acquisition, maintaining a frame rate of 40 to 90 frames/second at a stable heart rate is key. Clear endocardial and epicardial definition (seen throughout the cardiac cycle) is required to ensure adequate segmental tracking during systole and diastole. Markers are placed in each of the respective basal and apical regions, utilising automated tracking where possible to maintain high reproducibility Region of interest should be manipulated as required to fit the myocardium. Automated tracking should also be combined with a visual assessment of tracking in each view across the whole region of interest including the endocardial and epicardial border. If more than two segments in any one view are not adequately tracked, the average GLS should not be calculated. When the tracking of all myocardial segments is not feasible, but tracking of A4C segments is satisfactory, calculation of the A4C GLS should be performed. The method used should be made clear in the report LV GLS in cardiac amyloid is generally impaired and worse at the base and mid-ventricular regions when compared with the apex [55]. Apical sparing on the bullseye plot an apex:base ratio of > 2.1 helps distinguish cardiac amyloidosis from other causes of LV hypertrophy (such as hypertension, Fabry disease, Friedreich’s ataxia)	
TDI velocities	A4C,	PW TDI Units: cm/s	Systolic (Sm), early (e′) and atrial (a′) relaxation velocities at anterolateral, inferoseptal, inferior and anterior walls in expiration Reduced systolic velocity Reductions in TDI systolic and diastolic indices typically occur earlier in the natural history of the amyloid disease process than traditional echocardiographic measures, and may be a subclinical marker when this condition is suspected Typically a restrictive filling pattern with low e′, E/A > > 1, E/e′ (average of septal and lateral mitral annulus) > 13 is observed in cardiac amyloidosis Earlier in the natural history of restrictive disease, abnormalities of mitral annular PW TDI may correspond to the grade 1 or 2 categories of diastolic dysfunction	
LV diastolic function	A4C	2D CW Doppler PW Doppler TDIs	Increased LV filling pressure and restrictive mitral pattern with E/A ratio > 2 characterises the restrictive phenotype Diastolic parameters tend to be markedly abnormal in cardiac amyloidosis due to stiffening of the myocardium secondary to amyloid infiltration. TDI velocity at mitral annulus (e′) are usually < 6 cm/s, with bi-atrial dilatation and systolic blunting of PVD suggesting high filling pressures in the absence of significant MR [56]. E/e′ ratio becomes abnormal in early stages of amyloidosis (average of septal and lateral mitral annulus > 13)	
LVOT diameter, LVOT VTI for calculation of stroke volume	PLAX,A5C	2D, PW Doppler Units: LVOTd (mm), LVOT VTI, cm^2^; stroke volume, mL; stroke volume index, mL/m^2^	The LVOT diameter should be measured from the parasternal long-axis window immediately below the insertion of the aortic cusps, using an inner-edge to inner-edge methodology (not the apical windows). This recommendation differs from a historical practice whereby the LVOT has often been measured up to 1 cm below the point of insertion of the cusps Sweep speed 50–100 mm/s. Trace around modal velocity. Average three tracings in sinus rhythm (not shown) with a minimum of 5–10 consecutive beats for patients in AF. The PW Doppler sample volume should initially be placed at the level of the aortic valve, which will usually result in aliasing. It should then be slowly moved apically Please refer to the BSE aortic stenosis guidelines [45]	
Left ventricular outflow tract (LVOT) or intra-cavity obstruction	A5C, A3C	CW Doppler (or PW with HPRF as a significant gradient will alias on PW Doppler) Sampling PW Doppler throughout the LV cavity is a useful tool to pinpoint the exact location of obstruction if unclear on colour Units: mmHg	Assess obstruction gradients at rest and with Valsalva manoeuvre. Utilise colour flow mapping to locate areas of turbulent flow within the LV. Align CW Doppler through entire turbulent colour flow for peak obstruction velocity A peak gradient of ≥ 30 mmHg is considered significant. Record in report conclusion Although this is a less common feature in cardiac amyloid, it is important to identify to help distinguish from HCM	
*Valves*				
Thickening of aortic valve (AV), mitral valve (MV) and tricuspid valve (TV)	PLAX, RV inflow, SAX, A4C, A2C, A3C	2D Units: N/A	Visually assess the degree of thickening of the MV and AV leaflets using normal, mild, moderate, severe thickening statements to describe the valves. Although this is non-specific to cardiac amyloidosis, it is often an accompanying echo feature	
Mitral valve inflow	A4C	PW Units: cm/s	Place PW sample volume at the MV leaflet tips in the A4C view. Measure E wave, A wave, deceleration time, E/A ratio Restrictive MV filling pattern, is a classic feature of cardiac amyloidosis, but also indicates advanced stages of the disease High E/A ratio is often seen because of restrictive pathophysiology, but a reduced amplitude A wave may suggest poor atrial function and higher risk of thrombus formation Shortened mitral E deceleration time (restrictive filling pattern), high E/e′ ratio can be seen in cardiac amyloid, suggesting elevated LA pressures Please refer to the BSE diastolic function assessment guidelines	
Mitral regurgitation	PLAX, A4C, A3C	Colour flow mapping, CW	Mitral regurgitation quantification may be limited as the PISA dome may merge with turbulent LVOT flow. Mitral regurgitation secondary to SAM is mainly posteriorly directed. When quantitative assessment of MR is precluded by LVOTO, other indicators of MR severity should be considered. For example, an E velocity of < 1.3 m/s and an E/A ratio < 1 are strongly suggestive of non-severe MR	
Pulmonary venous Doppler	A4C	Colour flow mapping, PW Units: cm/s	Measure peak systolic (S) velocity, peak diastolic (D) velocity, the S/D ratio, peak atrial reversal (Ar) velocity in late diastole and the duration of the Ar velocity In the apical 4-chamber view, superior angulation of the transducer and use of colour flow will help locate the pulmonary veins. This angle often brings the aorta into the visualised plane The right upper pulmonary vein is usually easiest to see and is next to the atrial septum. If the signal is weak, ask the patient to adopt a more supine position. Place the PW Doppler sample volume (1–3 mm) 1–2 cm into the right upper vein. Wall filter settings should be lowered (100–200 MHz). Aim to include clear visualisation of the atrial reversal velocity waveform. Measurements should be averaged over 3 cardiac cycles, at end expiration Additionally, measure A wave duration on transmitral inflow For the measurement of the mitral valve A wave duration, the PW Doppler sample should be placed at the level of the annulus rather than at the leaflet tips. This provides a cleaner signal for the start and end of the wave	
Tricuspid regurgitation (TR) and echo probability of pulmonary hypertension	RV inflow SAX A4C	CW Colour flow mapping Units: Vmax m/s PG mmHg	Perform CW Doppler and colour quantification for TR in all views where the TV is visualised. See BSE pulmonary hypertension guidelines for estimating probability of pulmonary hypertension [57]	
*Atria*				
Left atrium (LA) biplane volume	A4C, A2C	2D biplane volume using independent A4C and A2C views Units: mL mL/m^2^	LA volume should be obtained from apical 4- and 2-chamber windows (separated by 60° of rotation), optimised for LA assessment, using the biplane Simpson’s method. Maximal LA volume should be obtained from the frame immediately prior to mitral valve opening. Values should be reported after indexing for BSA Trace the inner aspect of the left atrial wall. At the mitral valve level, the contour is closed by a straight line between along the plane of the mitral valve annulus. Exclude left atrial appendage and pulmonary veins	
RA	A4C	2D Units: cm cm/m^2^	RA area should be obtained from the apical 4 chamber window via an optimised RA assessment Measure at the end of ventricular systole on the frame just prior to tricuspid valve opening. Trace the RA from the plane of the TV annulus along the interatrial septum, superior and lateral walls of RA. At the tricuspid valve level, the contour is closed with a straight line between the TV annulus. Exclude the superior vena cava. Be careful not to falsely underestimate the area if device leads or Chiari network are present	
*Right ventricle (RV)*				
Right ventricular hypertrophy (RVH)	PLAX Subcostal	2D Units: mm	Freeze the PLAX or subcostal view of the RV free wall, scroll to end diastole and calliper the RV wall thickness. For best visualisation, zoom on RV free wall Coexisting right ventricular free wall hypertrophy strongly suggests an infiltrative cardiomyopathy because these are rarely seen in association with true LV hypertrophy	
RV size			The RV-focused view should be used to generate all apically acquired RV size and function quantification from a modified A4C view Freeze modified A4C view, scroll to end-diastole and calliper RVD1, RVD2 and RVD3 measurements. RVDI is measured at the maximal transverse diameter in the basal one third of the RV. RVD2 is measured at the level of the LV papillary muscles. Lastly, RVD3 length is callipered from the plane of the tricuspid annulus to the RV apex Typically, cardiac amyloidosis patients have normal RV end-diastolic dimensions	
RV systolic function – Tricuspid annular plane systolic excursion (TAPSE)	A4C	M-mode Units: mm	This is an angle dependent measurement and therefore it is important to align the M-Mode cursor along the direction of the lateral tricuspid annulus. Measure total excursion of the tricuspid annulus. The measurement should be a vertical line as shown in the figure, using the leading-edge to leading-edge technique Reduced TAPSE is an early indicator of cardiac involvement in patients with systemic AL amyloidosis	
RV systolic function—TDI	A4C	PW TDI Units: cm/s	PW tissue Doppler RV s′ wave measurement taken at the lateral tricuspid annulus in systole. Systolic velocity is measured by callipering the first upward velocity after the R wave It is important to ensure the basal RV free wall segment and the lateral tricuspid annulus are aligned with the Doppler cursor to avoid velocity underestimation. RV s′ is closely correlated with TAPSE, and these two measures should be concordant if measured correctly. A disadvantage of this measure is that it assumes that the function of a single segment represents the function of the entire ventricle RV indices are similar for males and females and do not vary significantly according to age Reduced RV TDI indices are an early indicator of cardiac involvement in patients with systemic AL amyloidosis	
RV systolic function – Fractional Area Change (FAC)		2D Units: EDA cm^2^ EDAi cm^2^/m^2^ ESA cm^2^ ESAi cm^2^/m^2^ FAC %	Assessment of fractional area change (FAC) is obtained from the RV-focussed A4C window (4). A contour is traced from the lateral tricuspid annulus along the free wall to the apex, and back along the interventricular septum to the medial tricuspid valve annulus. This is undertaken at end-diastole and the area indexed to BSA. This process can be repeated in end-systole to derive the FAC. Be careful to exclude the RV moderator band and muscle bundles	
*Other echo features of cardiac amyloidosis*				
Interatrial (IAS) thickening	SAX, A4C, Subcost-al	2D	Thickening of the inter-atrial septum (> 0.5 cm) can occur as a result of amyloid deposition and is a characteristic feature of cardiac amyloidosis	
Pericardial effusion	PLAX, SAX, A4C, A2C, A3C, Subcost-al	2D M-mode PW Doppler	Pericardial effusion is seen in more than 50% of cardiac amyloid patients [58]. Although this is non-specific to cardiac amyloidosis, it is often an accompanying echo feature Standard views and measurements should be acquired to determine the size, location and haemodynamic significance of the pericardial effusion [59]	

The timing of ventricular end diastole is taken as the frame before the mitral valve closes. Surrogates for this are the frame with the largest LV cavity size (diameter or volume), the start of the ECG QRS complex, or the ECG R-wave (a common trigger for analysis software).

The timing of ventricular end systole is taken as the frame where the aortic valve initially closes. This coincides with a closure click on the pulsed-wave Doppler tracing of aortic valve flow. When obtaining images from the apical 2- or 4-chamber views, end-systole is defined as the frame prior to mitral valve opening.

## Minimum dataset for TTE in cardiac amyloidosis

**Table:** 

Structure and function	Measurement
LV dimensions, and relative wall thickness	End-diastolic dimension (mm)
	End-systolic dimension (mm)
	Inter-ventricular septal thickness in diastole (mm)
	Left ventricular posterior wall thickness in diastole (mm)
	Relative wall thickness (RWT = IVSd + PWd / LVIDd)
LV wall thickness in short axis view	Septum at basal level, papillary muscle level and apex level (mm)
	Anterior wall at basal level, papillary muscle level and apex level (mm)
	Lateral wall at basal level, papillary muscle level and apex level (mm)
	Inferior wall at basal level, papillary muscle level and apex level (mm)
LV volumes	End-diastolic volume (mL), indexed to body surface area (mL/m^2^)
	End-systolic volume (mL), indexed to body surface area (mL/m^2^)
	Stroke volume (mL)
LV systolic function	Ejection fraction by Simpson’s Biplane (%)
	Ejection fraction by visual assessment (%)
	Global longitudinal strain (%)
	Presence or absence of apical longitudinal strain sparing pattern
Tissue Doppler Imaging	Anterolateral annulus (s′, e′, a′; cm/s)
	Inferoseptal annulus (s′, e′, a′; cm/s)
LV diastolic function	MV inflow PW Doppler (m/s)
	e′ and a′ TDI velocities (lateral and septal)
	LA biplane volume (mL)
	Pulmonary venous Doppler (m/s)
LVOT or intra-cavity obstruction (defining which)	Resting (mmHg)
	Valsalva (mmHg)
Mitral valve inflow Doppler	E wave (m/s)
	A wave (m/s) and A wave duration (ms)
	Deceleration time (ms)
Pulmonary venous Doppler	Systolic wave (cm/s)
	Diastolic wave (cm/s)
	Ar wave (m/s) and Ar duration (ms)
Mitral regurgitation	Severity
	Mechanism
	Direction of jet
Atria	LA biplane volume indexed to BSA (mL/m^2^)
	RA area indexed to BSA (cm^2^/m^2^)
RV	RV cavity size
	RV hypertrophy (mm)
	RV systolic function (TAPSE mm, TDI S cm/s, FAC %)
Tricuspid regurgitation and inferior vena cava	Severity
	Probability of pulmonary hypertension
	Inferior vena cava, size and collapse response
Inter-atrial septum	Degree of thickening (> 0.5 cm)
Effusions	Pericardial – size (cm), location and haemodynamic significance
	Pleural effusion
	Ascites

## Data Availability

Not applicable.
